# The influence of gender and ethnicity on facemasks and respiratory protective equipment fit: a systematic review and meta-analysis

**DOI:** 10.1136/bmjgh-2021-005537

**Published:** 2021-11-11

**Authors:** Jagrati Chopra, Nkemjika Abiakam, Hansung Kim, Cheryl Metcalf, Peter Worsley, Ying Cheong

**Affiliations:** 1 Scool of Human Development and Health, Faculty of Medicine, University of Southampton, Southampton, UK; 2 School of Health Sciences, Faculty of Environmental & Life Sciences, University of Southampton, Southampton, UK; 3 School of Electronics and Computer Science, University of Southampton, Southampton, UK; 4 Complete Fertility Southampton, Princess Anne Hospital, Southampton, UK

**Keywords:** COVID-19, respiratory infections, prevention strategies, health policies and all other topics

## Abstract

**Introduction:**

Black, Asian and minority ethnic (BAME) people are disproportionately affected by COVID-19. Respiratory protective equipment (RPE) has conventionally been developed for a predominantly white male population that does not represent the healthcare workforce. The literature was reviewed to determine the protection offered to female and BAME users.

**Methods:**

Five databases were searched. Eligible studies related to respirator fit in the context of anthropometrics, gender and ethnicity. Meta-analysis was performed for gender-based anthropometric differences. A priori protocol registration was not performed.

**Results:**

32 studies were included and anthropometric data was extracted from 15 studies. Meta-analysis revealed 14 anthropometric measurements were significantly smaller for females. Mean differences ranged from 0.37 mm to 22.05 mm. Gender-based anthropometric differences did not always translate to lower fit factor scores, with 12 studies reporting worse performance and fit for females and 10 reporting no gender effect. No studies provided disaggregate anthropometric data by ethnic group. Pass rates (PR) were low or moderate in 12 BAME or mixed-ethnicity cohorts. 14 studies reported associations between facial dimensions (FD) and respirator fit. Three comparative studies showed lower PR among selective BAME people. 18 studies reported RPE performance differed with model and design. Most studies did not prespecify inclusion/exclusion criteria. Small sample size and lack of justification or power calculations was a concern. Significant heterogeneity in study designs limited comparisons, particularly relating to respirator selection or availability and defining study outcomes relating to RPE performance.

**Conclusion:**

The literature reports on largely Caucasian or single ethnic populations, and BAME people remain under-represented, limiting comparisons between ethnic groups. Facial anthropometrics vary between gender and likely between ethnicity, which may contribute to lower PR among females and ethnic minorities, particularly Asians. There is a need for studies including a broader spectrum of ethnicities and for consideration of female and BAME users during RPE development.

**Key questions**

**What is already known?**

- For respirators to provide respiratory protection they must fit the user well, and this is determined by ‘fit-testing’—a process of trialling successive facemasks until one is identified which provides a good seal, and thus the user passes ‘fit-testing’.
- Pass rates (PR) are the proportion of participants that pass ‘fit-testing’ and are successful at identifying a facemask that fits.
- Several factors may affect respirator fit and performance; anthropometric influences are relatively well studied and described but the association of gender and ethnicity is disputed.
- The current COVID-19 pandemic is disproportionately affecting black, Asian and minority ethnics (BAME) healthcare workers most at risk and appropriately fitting respiratory protective equipment (RPE) is paramount.

**Key questions**

**What are the new findings?**

- Females have smaller facial measurements (3–15 studies) but gender-based differences in anthropometrics and lower PR are not always correlated with lower fit factor scores.
- Reporting of ethnicity-based differences in anthropometrics and RPE performance is limited.
- Gender was associated with RPE performance in 12 studies and fit test PR were greater for males in 8 studies.
- Overall PR were low or moderate for 12 studies of non-white cohorts.
- Female and BAME healthcare workers may experience difficulty in identifying respirators that offers adequate protection, requiring multiple fit-testing attempts.
- BAME people remain under-represented in the literature when evaluating RPE performance. Inclusivity of BAME people is needed in respirator design, fit-testing and research.

**What do the new findings imply?**

- Meta-analysis revealed 14 standardised anthropometric measurements were significantly smaller for females.
- Mean differences in measurements ranged from 0.37 mm for the smallest dimension (nasal root breath) to 22.05 mm for the greatest dimension (bitragion-menton arc).
- Meta-analysis of anthropometrics between ethnicity or of RPE performance outcomes was not possible due to reporting and study heterogeneity.
- There are limitations to the included studies, namely small sample size (n<50), inconsistency of RPE tested across participant cohorts, and risk of bias assessment showed most studies did not prespecify inclusion/exclusion criteria.
- Significant heterogeneity in study designs limits direct comparison.
- Including only English language studies is a significant limitation considering the focus of this review and inclusion of Chinese records in particular may affect results significantly.

## Introduction

There is growing evidence that black, Asian and minority ethnic (BAME) people are disproportionally affected by SARS-CoV-2 (COVID-19).^1–5^ Indeed, data from the UK-based Office for National Statistics demonstrates COVID-19 related death rates in BAME communities are four times higher compared with those of white ethnicity.^6^ BAME people comprise only 14% of the population in the UK, yet account for 34% of COVID-19-related admissions to intensive care and 35% of deaths.^7 8^ Similar trends are seen internationally.^9–11^ BAME people comprise a large proportion of workers in essential services,^12^ including healthcare, and their over-representation among patients affected by COVID-19 is a growing concern. Among National Health Service (NHS) staff, 63% of COVID-related deaths are of BAME people even though they represent only 20% of the NHS workforce.^13 14^ The effect is likely multifactorial,^4 5^ and addressing these ethnic inequalities requires efforts in various aspects, including effective personal protection equipment (PPE) in the workplace.

**Figure 1:**
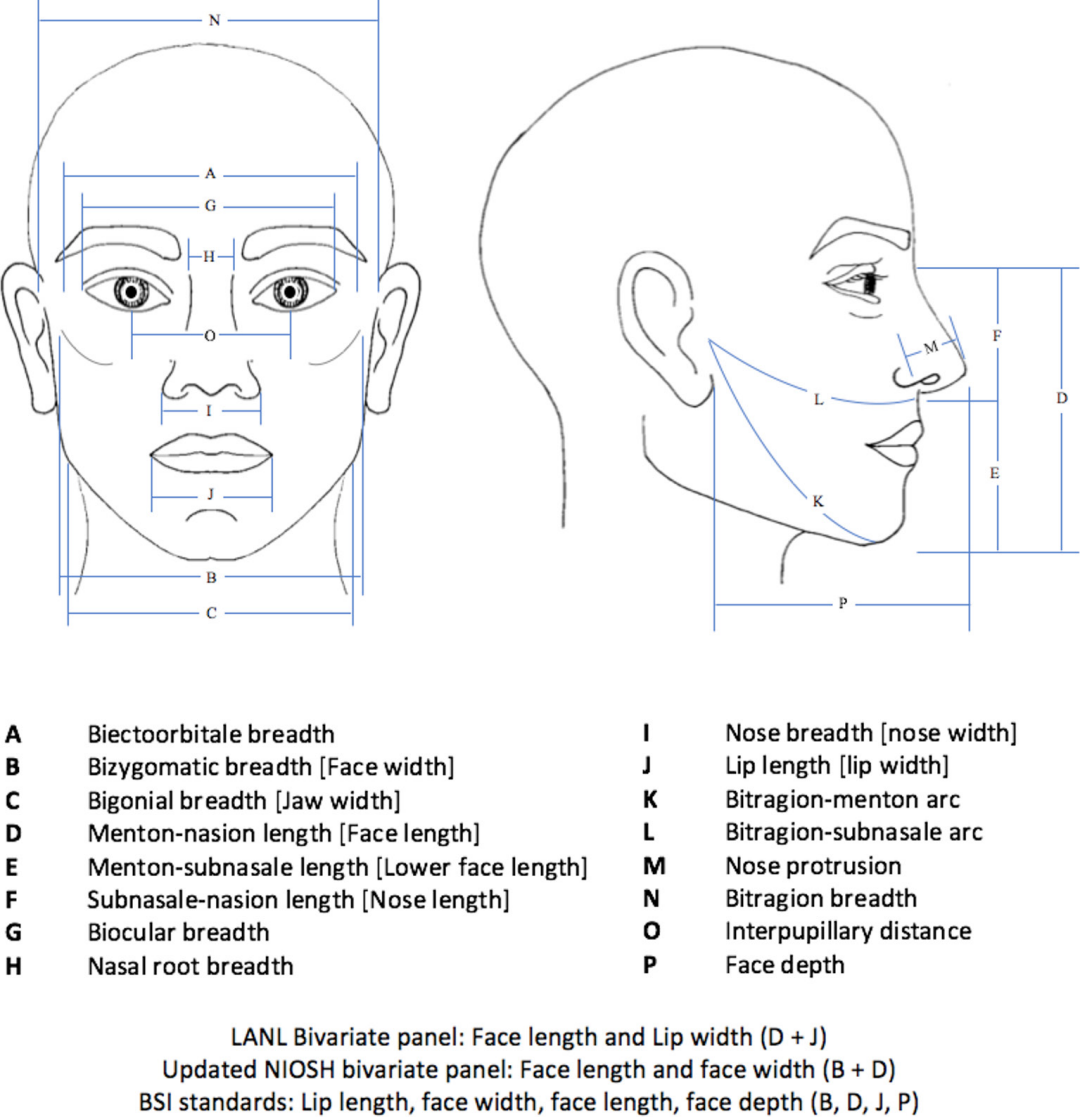
Anthropometric measurements.

Respiratory protective equipment (RPE) is vital in the prevention of nosocomial viral transmission. Systematic reviews and meta-analyses demonstrate the use of masks can reduce the risk of respiratory virus infection by 80%, suggesting mask use offers significant protection against transmission of respiratory viruses such as influenza, SARS and COVID-19.^15^ In the context of COVID-19, mask use has been shown to reduce the risk of infection by nearly 70% among healthcare workers, highlighting the importance of RPE in the current pandemic.^16^ European and American safety regulatory bodies such as the Occupational Safety and Health Administration (OSHA) or Health and Safety Executive mandate RPE must meet certification requirements, such as those developed by the National Institute for Occupational Safety and Health (NIOSH), International Organization for Standardization (ISO) or British Standards Institution (BSI).^17–19^ Certification requires respirators to be fit-tested on participants from a respirator fit test panel (RFTP) comprising subjects with facial sizes representative of the user population. Historically, sizing and respirator certification has been based on the Los Alamos National Laboratory (LANL) standardised adult head shape panels, developed in the 1960s using a US Air Force (USAF) Anthropometry Survey of predominantly white male military personnel.^20^ The bivariate RFTP referenced for half-mask respirators uses two facial measurements—face length and lip length (figure 1). With evolving population demographics such as changing body shape and increasing female and BAME representation, the USAF data is no longer reflective of the current American workers.^21^ Therefore, NIOSH created a novel anthropometric database. This has been used to update the bivariate panel to include face length and face width as well as identify 10 facial dimensions (FD) most relevant to respirator fit, which defines the principal component analysis model.^22^ In the UK, BSI standards have been based on the 50th percentile of four dimensions (face length, face width, face depth and mouth width) of the adult white male face shape (figure 1).^23^ More recent panels have included a more ethnically diverse sample group.

Fit testing is used to determine if the facial fit of a respirator is free of significant inward leak. Both qualitative fit test (QLFT) and quantitative fit test (QNFT) are recommended.^19 24^ QLFT uses one’s olfactory or taste response to an aerosolised solution. QNFT measures the ratio of external aerosol concentration to internal aerosol concentration, to produce a fit factor (FF) score. Definitions and standards have evolved over time, but currently OSHA recommends a QNFT FF score of 100 affords the user adequate protection and is equivalent to a successful QLFT.^24^ Suboptimal fit compromises respiratory protection and can be damaging to underlying skin.^25^

The relationship between FD and RPE shape determines RPE fit. FD vary significantly between genders, ethnicities and with age,^26^ as well on an individual basis. These may influence RPE fit and there is already some, although mixed, evidence that RPE protection varies with gender-based differences in facial dimension.^27–29^ Certainly, studies of BAME cohorts have yielded particularly low success rates of fit-testing, and similar trends are seen among healthcare workers.^28–30^ These findings may be important in respirator design and manufacturing processes. While newer RFTPs may be more diverse, they are not necessarily representative of healthcare workers (HCWs) or BAME people. There is growing concern that RPE in current use is inadequate at protecting female staff and those from at-risk BAME communities.^31^ The objectives of this systematic review were (1) to compare the anthropometric measurements of users across gender and ethnic groups and (2) to assess the effects of FD, gender and ethnicity on RPE fit and effectiveness as measured by fit-test FF scores, fit-test pass rates (PR) or inward leakage.

## Methods

The systematic review was conducted following the Preferred Reporting Items for Systematic Reviews and Meta-Analyses (PRISMA) guidelines.^32^ The PRISMA checklist is available in online supplemental appendix 1. A protocol for the review was defined, including inclusion and exclusion criteria but a priori protocol registration was not performed.

10.1136/bmjgh-2021-005537.supp1 Supplementary data

### Search strategy

A literature search was conducted using Embase and Medline via Ovid, PubMed, Scopus and Web of Science in April 2021. The search strategy (online supplemental appendix 2) included key terms relating to respirators, face masks or PPE, respirator fit, FD or facial anthropometrics and race or ethnicity. Gender anthropometrics and differences between sexes were found to be discussed in most studies, therefore gender search terms were not applied as these restricted search results. Reference lists of included papers were also screened. Only human studies, reported in English were included and no time restrictions were applied.

10.1136/bmjgh-2021-005537.supp2 Supplementary data

### Study selection and eligibility

Two authors independently screened the search results for relevance based on title and abstract, and unrelated studies were excluded. Subsequently, both authors reviewed full texts to identify studies meeting the inclusion criteria: human studies of any age/gender/ethnicity, assessing half or quarter size filtering facepiece respirators meeting N95/PPF3 standards. Studies pertaining to full-facepiece masks were excluded as these likely relate to different FD. Both disposable or reusable RPE was accepted regardless of brand, design, models and sizes. Studies relating to qualitative or quantitative fit-testing were eligible. Outcomes related to fit-test FF scores, fit-test PR or inward leak in the context of anthropometrics, gender and/or ethnicity. No restriction for setting were applied nor to participant characteristics such as occupation, ethnicity, race, gender or age. Studies not assessing the effect of at least one of, anthropometrics, gender or ethnicity, were excluded. Non-English language studies were excluded. Findings were compared and differences were addressed by re-review and discussion until a consensus was reached.

### Outcomes

The outcomes of this review were to compare the anthropometric measurements of users across gender and ethnic groups and assess the effect of FD, gender and ethnicity on RPE fit and effectiveness as measured by fit-test FF scores, fit-test PR or inward leakage.

### Data extraction

An initial data extraction pro-forma was piloted on a small number of records, modified as required and confirmed. Data extracted related to study characteristics and outcomes, including study design, study population, participant characteristics (age, gender distribution, race distribution), method of FD measurement, anthropometrics data, RPE type, fit-testing protocol, and outcome measures of differences in anthropometrics and in RPE fit. For meta-analysis, we intended to collate data on anthropometric measurements for gender and ethnic groups as well as disaggregated group FF scores and PR.

### Analysis

For systematic review, variables including FD, gender and ethnicity were organised into tables and described qualitatively. Association of variables FD, gender and ethnicity with RPE fit were summarised. Limitations and implications for this review are discussed.

Facial measurement means and associated SD were extracted where possible and a meta-analysis was performed for gender-based anthropometrics. Standardised methodologies for anthropometric measurements were employed by included studies and therefore sufficiently similar for meta-analysis. A random-effects meta-analysis was performed using RevMan.^33^ Statistical heterogeneity was assessed by the measure of I^2^. For facial measurements where I^2^ indicated substantial heterogeneity (>50%), study methods were reviewed for possible explanations. Studies were assessed for clinical and methodological heterogeneity to identify any outlying studies conflicting with the remaining studies across the 14 anthropometrics. Sensitivity analysis was conducted to determine whether the gender-based differences in anthropometrics are robust. Attempts were made to identify studies contributing to heterogeneity for exclusion. Anthropometrics were suspected to differ between ethnicities, therefore results were reviewed to identify groups of studies with conflicting results based on ethnicity for subgroup analysis.

Disaggregated anthropometric data was not available to allow for ethnicity-based FD comparisons. Due to heterogeneity in study design, outcome measures and reporting, meta-analysis could not be conducted for RPE performance.

### Risk of bias assessment

The National Heart, Lung and Blood Institute (NHRBI) study quality assessment tools for observational cohort and cross-sectional studies^34^ has previously been adapted^35^ to assess the quality of studies in the context of anthropometric measurements between gender groups. The NHRBI tool was similarly modified and applied to the studies included in this systematic review based on available guidance from the NHRBI tool.

### Patient and public involvement

This research does not directly include patient or public involvement. The aims and questions are informed by national and international experiences of female and BAME HCWs in using RPE during the ongoing pandemic.

## Results

### Literature search results

**Figure 2:**
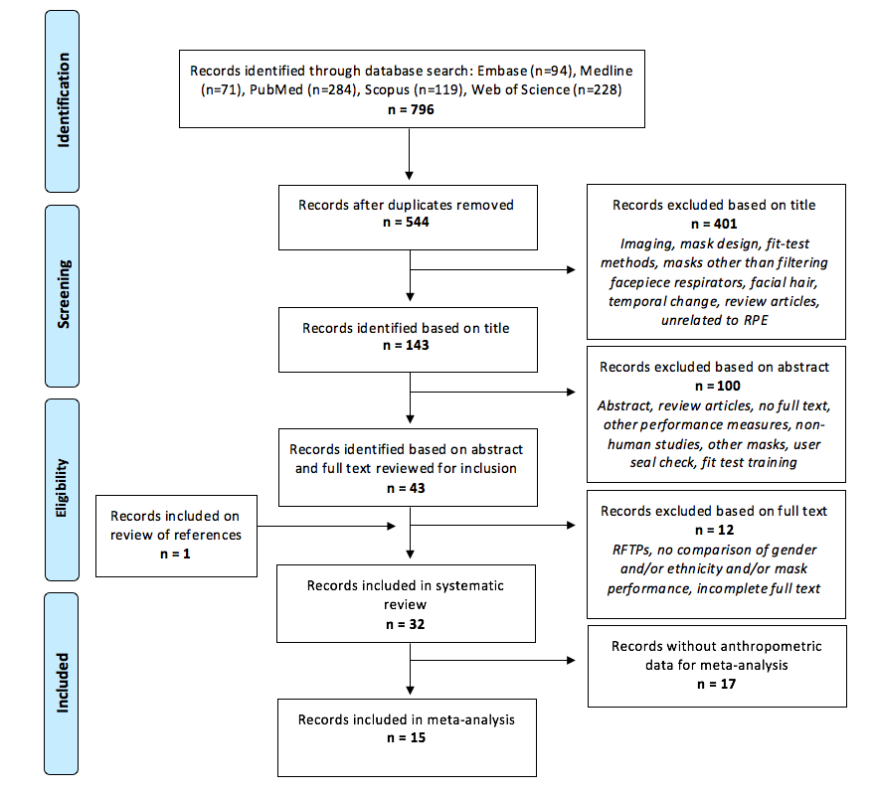
Preferred Reporting Items for Systematic reviews and Meta-Analyses flow diagram detailing study selection. RFTP, respirator fit test panel; RPE, respiratory protective equipment.

Search of the five databases yielded 796 records, with 544 remaining after excluding duplicates (figure 2). Of these, 401 studies were excluded based on title alone and 100 studies based on abstract. These were either unrelated to RPE or pertained to mask-design, methods of fit-testing and other predictors such as facial hair and temporal changes. Review articles and conference papers were also excluded. Full texts were reviewed for the remaining 43 records and a further 12 articles were excluded.^36–47^ Further detail of reasons for exclusion are shown in online supplemental appendix 3. One additional study was included from screening of references. Therefore 32 articles were identified as eligible for inclusion.^27–30 48–75^ Publication year ranged from 1982 to 2021, and all publications were in English. Most studies were published in non-medical journals, largely relating to occupational, industrial or environmental hygiene, ergonomics or health and safety fields. Finally, 15 studies reported anthropometric measurements for meta-analysis.^27 29 49 51 53–55 60 61 63 65–67 71 73^

10.1136/bmjgh-2021-005537.supp3 Supplementary data

### Study characteristics

Study characteristics are presented in table 1. The 32 included studies yielded a total of 10 658 participants, of which 33% were male and 60% female, with 8% being unreported. Four studies included a Caucasian population,^48 55 60 69^ five studied a Korean population,^29 52 53 65 68^ two studied a Chinese population,^63 72^ three studied an Iranian population,^67 71 74^ one studied a Taiwanese population^66^ and one studied a Latino migrant workers population.^62^ Eight studies had populations of mixed ethnicity,^27 28 50 51 57 59 61 75^ with the predominant group being Caucasian or black/African. Ethnicity was not reported for eight studies, which were based in Australia, France, Spain, UK and the USA.^30 49 54 56 58 64 70 73^ The distribution of participant ethnicities is shown in online supplemental appendix 4. Participants included HCWs, university students and staff or civilian workers from surrounding communities.

10.1136/bmjgh-2021-005537.supp4 Supplementary data

**Table 1:** Study characteristics

Study, country	No. of participants (% male)	Age range (years), (mean*)	Population	Population ethnicity† (%)	RPE type Number of brands/models/sizes (RPE tested per user)****	No. of facial dimensions	RPE fit measure (guidelines/standards) and outcome
Liau *et al*, USA^48^	190 (100%)	N/A	Laboratory employees	Caucasian	Reusable half mask 4 brands, 10 sizes total	7	QNFT Protection factor
Gross and Horstman, USA^49^	121 (50%)	(37.5)	Community members	N/A	Reusable half masks 3 brands (2), 3 sizes each	10‡	QNFT§ (ANSI) Fit factor, pass rates
Oestenstad *et al*, USA^50^	73 (53%)	21–50 (30.6)	University student, staff and faculty	White (68%) Black (12%) Asian (12%) Other¶ (7%)**	Reusable half mask 1 brand, 3 sizes	12	QNFT (ANSI) Leak shape, size and distribution†† Fit factor
Oestenstad and Perkins, USA^27^	68 (56%)	21–50 (30.4)	University students and staff	White (69%) Black (13%) Asian (10%) Hispanic (4%) Asian Indian (3%)**	Reusable half mask 1 brand, 3 sizes	12‡	QNFT†††† (ANSI) Fit factor
Brazile *et al*, USA^51^	186 (49%)	N/A	Community members, university students	White (35%) African (31%) Mexican (33%)	Reusable half mask 1 brand, 3 sizes	14‡	QNFT§ (ANSI) Fit factor, pass rates
Han, South Korea^52^	778 (52%)	20–55	Industrial workers, university students	Korean	Reusable quarter mask 3 brands (3)	2	QNFT‡‡(ANSI) Fit factor, pass rates
Han and Choi, South Korea^29^	150 (75%)	20–55	Community members, university students	Korean	Reusable half mask 3 brands (3), 1 size (M)	10‡	QNFT‡‡(ANSI/OSHA) Fit factor, pass rates
Kim *et al*, South Korea^53^	110 (64%)	N/A	University students	Korean	Reusable quarter mask 3 brands (3), 1 size (M)	12‡	QNFT§‡‡ (ANSI/OSHA) Fit factor, pass rates
Zhuang *et al*, USA^54^	32 (45%)	N/A	N/A	N/A	Disposable N95 half mask 18 models (18), 1–3 sizes	12‡	QNFT SWPF
Oestenstad *et al*, USA^55^	41 (51%)	20–55 (30)	Institute student and staff	Caucasian	Reusable half mask 3 brands (3), >1 size	12‡	QNFT Fit factor
McMahon *et al*, Canada^56^	1295 (24%)	19–71	Healthcare workers	N/A	Disposable N95 half masks 3 brands§§, 6 models	N/A	QLFT Pass rates
Zhuang *et al*, USA^57^	30 (43%)	N/A	Community members	While (90%) Black (33%) Asian (66%)	Disposable and reusable half masks 4 models (4), 3 sizes	3	QNFT‡‡ Fit factor, pass rates
Winter *et al*, Australia^58^	50 (N/A)	N/A	Healthcare workers	N/A	Disposable N95 half masks 2 brands, 3 models (3)	1	QLFT
Wilkinson *et al*, Australia^59^	5024¶¶ (21%)	Mode age group: 41–50	Healthcare workers	Aboriginals (0.9%) White (88.9%) East-Asian (5.7%) South/Central- Asian (3.5%) Other*** (1.0%)	Disposable P2/N95 half masks 3 brands§§, 1–2 sizes	N/A (overall face shape/size data collected)	QNFT‡‡ Pass rates
Oestenstad and Bartolucci, USA^60^	41 (51%)	20–55 (3)	University students and staff	Caucasian	Reusable half masks 3 brands (3)	12‡	Leak size, shape and distribution ††
Spies *et al*, South Africa^61^	29 (48%)	N/A	Research institute employees	African (45%) European (41%) Coloured††† (7%) Asian (7%)	Disposable P2 half mask 1 model, 1 size (M)	4‡‡‡ ‡	QNFT‡‡ (OSHA) Fit factor, pass rates
Ciotti *et al*, France^30^	50 (N/A)	N/A	Healthcare workers	N/A	Disposable PPF2 half masks 9 models (2-3)	N/A	QNFT‡‡ Fit factor, pass rates
Earle-Richardson *et al*, USA^62^	56 (88%)	15–81 (33.2)	Farmworkers	Latino	Disposable N95 and reusable half masks 4 brands, 7 models§§	N/A	QLFT (OSHA) Pass rates
Yu *et al*, China^63^	50 (52%)	Mean age (SD) 21.5 (2.2)	N/A	Chinese	Disposable N95 half masks 4 brands, 10 models (10), 2 sizes	21‡	QNFT‡‡ (OSHA) Fit factor, pass rates
Bergman *et al*, USA^64^	229 (N/A)	N/A	General population	N/A	Disposable N95 half masks 7 models§§, 1–2 sizes	13	QNFT‡‡ (OSHA) Fit factor, inward leak
Kim *et al*, South Korea^65^	49 (67%)	Mean age (SD) 23.0 (3.8)	Healthcare workers	Korean	Disposable N95 half masks 1 brand, 2 models, 3 sizes	7‡	QNFT‡‡ (OSHA) SWPF, fit factor, pass rates
Lin and Chen, Taiwan^66^	206 (49%)	21–30	Community members, university students	Taiwan	Disposable N95 half masks 3 models (3), 1 size	19‡	QLFT (OSHA)
Manganyi *et al*, South Africa^28^	562 (33%)	Mode age group: 19–30 years	Laboratory employees	African (65%) Asian (11%) Coloured§§§ (9%) White (14%)	Disposable N95/FFP2 half masks >2 brands (1) ¶¶¶, 2 sizes (S, M)	4	QNFT‡‡ (OSHA) Fit factor, pass rates
Honarbakhsh *et al*, Iran^67^	95 (33.5%)	N/A	Healthcare workers	Taiwanese	Disposable N95 half masks 3 models, 1 size	2‡	QLFT (OSHA) Pass rates
Huh *et al*, South Korea^68^	211 (51%)	Median 26 IQR 23–31	Military hospital volunteers	Korean	Disposable N95 half masks 3 brands, 4 models (4), 1–3 sizes	2	QNFT‡‡ (OSHA) Fit factor, pass rates
Foereland *et al*, Norway^69^	127 (88%)	18–65 (37)	Smelting industry workers	Norwegian	Disposable P3 half masks 4 brands, 14 models (≥5), 1 or 3 sizes	N/A	QNFT‡‡ (OSHA) Fit factor, pass rates
Winski *et al*, UK^70^	262 (90.5%)	N/A	General population	N/A	Disposable PPF3 half mask 1 model	3	QNFT‡‡ (BSIF) Fit factor, pass rates
Fakherpour *et al*, Iran^71^	62 (40%)	Mean age (SD) 23.45 (4.66)	University students	Iranian	Disposable N95/PPF2/FFP3 half masks 4 brands (4)	2‡	QLFT Pass rates
Zhang *et al*, China^72^	85 (36%)	Mean age (SD) 27 (4.4)	University students	Chinese	Disposable N95/FFP3 half masks 4 models (4), 1 size	8	QNFT‡‡ Fit factor, pass rates
De‐Yñigo‐Mojado *et al*, Spain^73^	74 (50%)	Mean age (SD) 34.31 (7.13)	Healthcare workers	N/A	Disposable FFP3 half masks 2 brands, 3 models	4‡	QNFT‡‡ Fit factor, pass rates
Fakherpour *et al*, Iran^74^	37 (32%)	Mean age (SD) 24.6 (4.2)	University volunteers	Iranian	Disposable N95/FFP2 half masks 15 brands, 20 models (20)	2	QNFT‡‡ Fit factor, pass rates
Williams *et al*, Australia^75^	96 (57%)	Mean age (SD) 42.3 (9.5)	Healthcare workers	South East Asian (26%) Other (74%)	Disposable N95 half mask 2–3 models§§, 2 sizes	N/A	QNFT‡‡ (OSHA) Fit factor, pass rates

*Unless otherwise stated.

†Ethnicity as reported by authors of respective studies. Efforts were made to determine ethnicity if not clearly reported.

‡Studies with anthropometric data reported for inclusion in meta-analysis.

§QNFT FF score of 10 used as equivalent to effective protection using particulate detector or condensation nuclei count (portacount) method.

¶Hispanics and Asian Indians.

**Data on ethnicity collected but no comparison made due to small numbers.

††RPE performance measured used fluorescent tracer.

‡‡QNFT FF score of 100 used as equivalent to effective protection using condensation nuclei count (portacount) method.

§§An initial respirator was selected. Once a successful fit test was obtained other models were not tested. In the event of failed testing, subsequent models were tested until fit-testing was passed.

¶¶Survey study design, with ‘no. of participants’ representing number of healthcare workers who responded to the questionnaire and were tested with the respirators. Percentage of males calculated number of questionnaires where participants supplied information on gender.

***Not reported.

†††Mixed European, African or Asian ancestry as per consensus referenced in the study.

‡‡‡Two measurements taken for all participants, two additional measurements taken on small proportion of participants.

§§§Mixed-race, combination of ethnic backgrounds including African, White, Khoisan, Indian and Malay.

¶¶¶Participants were tested using the type and size of mask used in the workplace at the time of study.

****Number of brands/models/sizes included in the study. (N) describes the number of masks tested per participant, where reported. Intra-study variability in number of masks tested per participant observed.

††††QNFT FF score of 1000 used as equivalent to effective protection using photometric method.

ANSI, American National Standards Institute; BSIF, British Safety Industry Federation; FF, fit factor; N/A, not available/reported; OSHA, Occupational Safety and Health Administration; PF, protection factor; PR, pass rates; QLFT, qualitative fit test; QNFT, quantitative fit test; RPE, respiratory protective equipment; SWPF, simulated work place protection factor.

Between 1 and 21 FD were measured by 26 studies in varying combinations^27–29 48–55 57 58 60 61 63–68 70–74^ and one study recorded overall face shape and size.^59^ The most frequently reported anthropometrics are shown in figure 1 which references standardised measurements from the US air force anthropometric report.^76^ Fit-testing protocols were in accordance with regulations relevant at the time of study, including ANSI and OSHA standards and in most studies involved quantitative measurement of FF using a PortaCount Plus. Six studies performed qualitative fit-testing^56 58 62 66 67 71^ and two assessed inward leak.^50 60^ The variety of RPE brands, models and sizes used and fit-testing methods are reported in table 1.

### Systematic review and meta-analysis findings

Study results were compared qualitatively. Comparisons of anthropometrics between gender and ethnicity groups are shown in table 2. Anthropometric data was available for meta-analysis from 15 studies.^27 29 49 51 53–55 60 61 63 65–67 71 73^ Mean differences and 95% CIs for 14 standardised anthropometric measurements are shown in table 3, with complete data and forest plots available in online supplemental appendix 5. A random-effects meta-analysis revealed all 14 anthropometric measurements were significantly smaller for females (p<0.05). Differences ranged from 0.37 mm for the smallest measurement (nasal root breadth) to 22.05 mm for the longest measurement bitragion-menton arc). Heterogeneity was substantial (I^2^>50%) for nine FD. Gender effect was in the opposite direction in one study, with greater face length and face width for females.^71^ Sensitivity analysis with exclusion of this study increased the mean difference between genders minimally and improved I^2^ by 10% for face length and 6% for face width. No specific study was identified to contribute substantially to heterogeneity across all 14 measurements. Therefore, no further studies were excluded for sensitivity analysis. Separation of studies by ethnicity did not improve I^2^ substantially but significantly reduced participant population, therefore subgroup analysis was not performed. Data for anthropometrics of ethnic groups were not available to meta-analyse. Effects of anthropometrics, gender and ethnicity on RPE fit are summarised in table 4, with complete data per study available in online supplemental appendix 6. Disaggregated data for FF scores and/or PR were not available and heterogeneity in study design and reporting hampered direct comparison of RPE fit outcomes between studies.

10.1136/bmjgh-2021-005537.supp5 Supplementary data

10.1136/bmjgh-2021-005537.supp6 Supplementary data

**Table 2:** Comparison of anthropometrics between gender and ethnicity groups

Studies, country	Comparison of anthropometric measurements		
	Between genders	Between ethnicities	To other populations/panels
Gross and Horstman, USA^49^	♀smaller dimensions for 11/12 FD	N/A	Comparable to US Air Force population
Oestenstad *et al*, USA^50^	N/A	No comparison made due to small sample size	Skewed distribution relative to LANL panel
Oestenstad and Perkins, USA^27^	♀smaller dimensions	No comparison made due to small sample size	Comparable to previous studies and US Air Force population
Brazile *et al*, USA^51^	♀smaller dimensions for 12/14 FD except binocular and NRB	Significant difference between ethnic groups except for FL	Comparable to previous studies and US Air Force population
Han, South Korea^52^	♀smaller dimensions separately screwed distribution of FD but with significant overlap	N/A	N/A
Han and Choi, South Korea^29^	♀smaller dimensions for all 10 FD	N/A	N/A
Kim *et al*, South Kore^53^	♀smaller dimensions for 11/12 FD except for NRB	N/A	Comparable to Korean cohorts. Different (smaller and wider faces) to American cohorts
Zhuang *et al*, USA^54^	♀ smaller dimension for 9/12 measurements except LW, NRB, NP	N/A	N/A
Oestenstad *et al*, USA^55^	♀smaller dimensions for 10/12 FD except LFL and NL	N/A	Comparable to previous studies
Wilkinson *et al*, Australia^59^	N/A	Facial characteristics were strongly associated with racial group	N/A
Spies *et al*, South Africa^61^	♀smaller and narrower dimensions	Comparison not made	Screwed distribution relative to LANL panel. Mean FD comparable to Korean and American cohorts but male FD different (smaller and wider) from American cohort
Yu *et al*, China^63^	♀smaller dimensions	N/A	Comparable to Chinese cohort. Different (smaller and wider) to American cohorts
Kim *et al*, South Korea^65^	♀smaller LW only	N/A	N/A
Manganyi *et al*, South Africa^28^	♀smaller dimensions	Asian♀: smaller dimensions African ♂: greater NRB	N/A
Lin and Chen, Taiwan^66^	♀smaller dimensions separately screwed distribution of FD	N/A	Screwed distribution relative to NIOSH panel. Difference to American cohorts (smaller)
Honarbakhsh *et al*, Iran^67^	N/A	N/A	Significant proportion outside RFTP. Different to South African, Korean and American cohorts (smaller FL and FW)
Fakherpour *et al*, Iran^71^	FD reported as similar but no comparison clearly reported	N/A	Skewed distribution relative to panel and significant proportion outside NIOSH RFTP
De‐Yñigo‐Mojado *et al*, Spain^73^	♀smaller dimensions	N/A	N/A

♀=female; ♂=male.

FD, facial dimensions; FL, face length; FW, face width; LANL, Los Alamos National Laboratory; LFL, lower face length; LW, lip width; N/A, not available/assessed or not reported; NIOSH, National Institute for Occupational Safety and Health; NL, nose length; NP, nose protrusion; NRB, nasal root breadth; RFTP, respirator fit test panel.

**Table 3:** Summary of anthropometric measurements and mean differences from meta-analysis

Outcome	Studies	Participants (n)	Male participants (n)	Female participants (n)	Mean difference (CI)
Biectoorbitale breadth	4	260	150	110	9.26 (7.54 to 10.97)
Bizygomatic breadth (face width)	15	1503	742	761	7.54 (6.80 to 8.27)
Bigonial breadth (jaw width)	8	834	468	366	6.75 (5.81 to 7.69)
Menton-nasion length (face length)	15	1503	742	761	7.82 (7.13 to 8.50)
Menton-subnasale length (lower face length)	9	727	409	318	5.26 (4.54 to 5.97)
Subnasale-nasion length (nose length)	9	973	541	432	3.64 (3.16 to 4.13)
Biocular breadth	4	260	150	110	3.87 (3.00 to 4.74)
Nasal root breadth	8	734	382	352	0.37 (0.12 to 0.61)
Nose width	12	1083	585	498	3.42 (3.06 to 3.78)
Lip length/width	13	1157	622	535	2.82 (2.36 to 3.28)
Bitragion-menton arc	9	884	494	390	22.05 (20.15 to 23.95)
Bitragion-subnasale arc	10	933	510	423	18.43 (16.70 to 20.16)
Nose protrusion	6	745	405	340	2.03 (1.65 to 2.40)
Interpupillary distance	3	288	141	147	2.70 (2.02 to 3.39)

**Table 4:** Summary of findings: association of variables facial dimensions, gender and ethnicity with RPE fit

Outcome	Studies assessing outcome	Summary of findings (n=number of studies)		
		Significant association	Weak association or mixed results	No association
Pass rates†	26	High (≥75%) overall user PR*: n=9. Moderate (50%–74%) overall user PR: n=4. Low (<50%) overall PR: n=4. Variable PR between gender and/or ethnic groups: n=9. Overall low or low-moderate PR in studies of non-white cohorts (n=12).		
Association between facial dimensions (FD) and fit**	25	n=14. FD association with fit: FW (n=7), FL (n=6), NRB (n=4), JW (n=4), LFL (n=3), NL (n=3), NP (n=3), NW (n=2), LW (n=2), BIOC (n=2), BECTO (n=2), BTMA (n=2). Facial size and shape categories associated with FF (n=6). Extremes in FW and FL or narrower faces associated with fit (n=2).	n=7. Association of some facial dimensions (FW, LW, JW NW, NP, NL, NRB) but low correlation coefficient, poor predictors of fit, explain small proportion of variability, small absolute differences or effect size.	n=4. No significant correlations. No relationship between facial size categories and fit.
Association between gender and fit‡	24	n=12. PR males>females (n=8). PR females>males (n=2). Gender differences in PR varied with model (n=1). Association of facial dimensions and leak site attributed to gender. Male gender is independent predictor for fit.	n=2. PR males>females for at least 1 RPE model but comparable overall with inclusion of multiple models.	n=10. No significant correlations. Comparable PR between genders. Fit not predicted by gender.
Association between ethnicity and fit†	5	n=2. Race specific models improve fit predictability. Lower PR for Asians, highest for Caucasians.	n=2. Lower PR for black females, No significant effect on FF. Lower PR and FF for Asian females, race did not predict FF.	n=1. Non-significant ethnicity-based variation in FF.
Association between mask factors and fit§	20	n=18. Variability in FF based on brand. Significant difference in FF/PR between brands (n=12). Influence of RPE on fit within facial size categories (n=2) or shape (n=1). RPE is determinant or predictor of fit (n=2).	n=1. FF associated with number of sizes and models, not RPE design.	n=1. Comparable PR between models.

*Overall user pass rates—percentage of participants successfully fit-testing on at least one RPE model.

†PR are reported as either (1) PR of users, as a percentage of participants who passed fit-testing on at least one respirator or (2) PR for RPE groups, as a percentage of participants who passed fit-testing for the respirator being tested.

‡RPE fit as measured by respective studies, including fit/protection factor (FF), simulated workplace protection factor (SWPF), inward leakage (IL), fit-testing PR (PR).

§Mask factors are reported as any differences in FF or PR relating to mask factors such design, model, brand, shape or size.

BECTO, biectoorbitale breadth; BIOC, biocular breadth; BTMA, bitragion-menton arc; FL, face length; FW, face width; JW, jaw width; LFL, lower face length/menton-subnasale length; LW, lip width; NL, nose length/subnasale-nasion length; NRB, nasal root breadth; NW, nose width; PR, pass rates; RPE, respiratory protective equipment.

### Qualitative synthesis

#### FD differ with gender

Gender-based anthropometrics were compared by 15 studies (table 2). Overall, 13 studies demonstrated gender differences, with smaller average female FD for most measurements.^27–29 49 51–55 61 63 66 73^ Female measurements were reported to range between 91.5% and 92.5% of the comparable male measurements although with significant overlap of 20%–50%.^49 53^ Some studies reported no gender differences for nasal root breadth,^51 53 54^ nose length,^55^ nose protrusion^54^ and lip width,^54^ lower face length^54^ and one reported greater smiling lip length for females.^49^ Meta-analysis demonstrated that all anthropometric were significantly smaller for females than males. Differences in nasal root breadth were minimal but still statistically significant (table 3).

#### FD differ with ethnicity

Ethnicity data was collected by six studies, of which two studies reported anthropometric data and between-group differences. An American study with participants from three ethnic groups found significant differences in all facial measurements, except face length.^51^ Interestingly, facial measurements were comparable to early studies comprising a largely Caucasian male population. A South African study including four ethnic groups also reported variation between ethnicities.^28^ Asian females had significantly smaller facial measurements and black males had greater nasal root breath measurements as compared with their white counterparts. An Australian survey collected information on overall facial shape and nose size/shape rather than anthropometric measurements and reported facial characteristics were strongly associated with racial group. The three remaining studies were unable to compare anthropometrics between ethnic groups due to small sample sizes.

Studies also drew comparisons between their cohorts and those of previous studies (table 2). Studies of various Asian populations reported significantly different FD compared with Caucasian cohorts, with generally smaller and wides faces. Korean participants had wider face width and nose breadth, narrower nasal root breadth and lip width.^53^ Chinese and Iranian participants had wider face width and shorter face length^63 67^ and Taiwanese participants had overall smaller faces.^66^ FD of males from an ethnically mixed South African cohort were also smaller and wider than for Caucasians.^61^ Several studies showed skewed distribution of participant FD compared with the American panel FD such that significant proportions of their cohorts lie outside RFTPs.^50 61 66 71^

#### Gender effects on RPE fit

Gender-based differences in anthropometrics have not consistently translated to a difference in FF (table 4). Of 24 studies comparing PR and/or FF scores between genders, 13 studies demonstrated significant gender effects. Of these, 11 studies reported higher fit-test failure rates and/or lower FF scores among females.^28 29 49 52 53 56 63 67 68 71 73^ Factors such as facial stubble which hamper RPE performance may reduce fit for males such that PR appear similar between genders, but comparison of only clean-shaven males yielded higher PR than for females.^28^ Gender was also reported to account for a higher proportion of variability in FF scores in analysis of variance. Association of FD and leak sites was mostly attributed to gender.^50^ Two studies did not compare PR but did demonstrate an association of gender-based FD with leak distribution and greater predictability of FF using gender specific models.^27 50^

In comparison, 11 studies reported no gender effects, with similar PR, no effect on FF score or no effect of gender on leak distribution/shape/sizes.^51 54 55 58–61 69 72 74 75^ One study reported mixed results with higher PR among males for two of three RPE models but comparable PR overall across all RPE models.^49^ A further study reported higher PR among female users.^73^ The variable effects of gender on RPE fit may be the result of differences in methodology. Study design was variable, with some studies assessing one model in multiple sizes, multiple models in one size or multiple models and sizes. For example, PR were higher for males than females for certain mask models, vice versa for others or comparable.^29 68 72^ Similarly, PR were higher among males when restricted to comparisons between individual mask models and introduction of multiple models improved overall female PR.^49^

### Ethnic effects on RPE FF scores

FF scores were compared between ethnic groups by only three studies (table 4). Differences in facial measurements between three American ethnic groups did not translate to significant differences in FF scores.^51^ A South African study demonstrated FF varied with ethnicity but was underpowered to detect significance of these differences.^61^ This is supported by a larger South African cross-sectional study which reported, while FF scores were lowest among Asians and variable between ethnicities, ethnicity was not a significant predictor for fit in the logistic regression analysis.^28^ A further study with ethnically mixed participants demonstrated FD-based predictability of FF scores improved with race specific models.^27^

### Higher fit-test failure rates in ethnic minority groups

Four studies revealed PR correlated with ethnicity. Among an evenly mixed cohort of Caucasian, African and Mexican Americans, PR were lowest among African American females.^51^ Both South African studies have demonstrated particularly low PR at 13.8% and 22% in their mixed cohorts of predominately BAME participants, using single model/size RPE and multiple brands/sizes RPE, respectively.^28 61^ In particular, the lowest PR were seen in Asian females.^28^ The largest study, an Australian survey, similarly reported the highest failure rates were among Asian HCWs and the highest PR were among white HCWs.^34^

Of studies assessing BAME cohorts, ten have reported particularly low PR with significant variability between RPE models. Studies of solely Chinese or Korean cohorts report low PR when assessing subgroups for gender and certain mask type. While some masks were associated with PR between 60% and 87%, others were successful for only 10%–30% of users.^29 52 53 63 66–68 71 72 74^ Chinese and Iranian studies even found some masks were ineffective for all of their participants.^63 67 74^ Masks that are a good fit for Caucasian Americans have been shown to provide adequate fit for only 41% of Latino workers.^62^ Additionally, two European studies demonstrate low PR among HWCs, suggesting current RPE may be inadequate, however, the ethnic distribution of these populations was not reported.^30 73^

### Mask factors affect RPE performance

A total of 20 studies compared FF and/or PR between different RPE brands and models; 17 studies demonstrated RPE performance differs significantly based on design.^28–30 52 53 55 57 59 63 64 66–69 71 72 74^ One study reported FF score varied with RPE brand for females only, with no correlation in the male group.^49^ A study assessing 18 RPE models however demonstrated the number of models and sizes available is associated with FF, rather than the RPE design itself.^54^

### Risk of bias within studies

Quality assessment is presented in table 5. The majority of studies fail to meet criteria three as inclusion and exclusion criteria were not prespecified. The majority of studies also do not provide sample size justifications or power calculations. However, many are still able to meet criteria four as they report on variance or effect estimates, as detailed by the NHLBI assessment tool. Of note, several studies do not meet criteria five as anthropometric data were not collected.

**Table 5:** Assessment for bias using modified National Heart, Lung and Blood Institute (NHRBI) study quality assessment tools

Studies	Criteria									
	1	2	3	4	5	6	7	8	9	10
Liau *et al*^48^	·	·	￮	·	◐	·	◐	·	·	·
Gross and Horstman^49^	·	·	￮	◐	·	·	·	·	◐	·
Oestenstad *et al*^50^	·	·	￮	·	·	·	·	·	·	·
Oestenstad and Perkins^27^	·	·	￮	·	·	·	·	·	·	·
Brazile *et al*^51^	·	·	￮	·	◐	·	·	·	·	·
Han^52^	·	·	￮	·	·	·	·	·	·	￮
Han and Choi^29^	·	·	￮	·	·	·	·	·	·	￮
Kim *et al*^53^	·	·	·	·	·	·	·	·	·	·
Zhuang *et al*^54^	·	￮	￮	·	·	◐	·	·	·	￮
Oestenstad *et al*^55^	·	·	·	·	·	·	·	·	·	·
McMahon *et al*^56^	·	·	·	￮	N/A	◐	·	·	·	￮
Zhuang *et al*^57^	·	·	￮	·	·	·	·	·	·	·
Winter *et al*^58^	·	·	·	￮	N/A	·	·	·	◐	·
Wilkinson *et al*^59^	·	·	￮	￮	N/A	◐	◐	·	·	·
Oestenstad and Bartolucci 60	·	·	￮	·	·	·	·	·	·	·
Spies *et al*^61^	·	·	·	￮	◐	·	·	·	◐	·
Ciotti *et al*^30^	·	·	￮	￮	N/A	·	·	·	·	·
Earle-Richardson *et al*^62^	·	·	￮	￮	N/A	◐	·	￮	·	·
Yu *et al*^63^	·	·	·	￮	·	·	·	·	·	￮
Bergman *et a*^64^	·	·	￮	￮	·	·	·	·	·	·
Kim *et al*^65^	·	·	￮	·	·	·	·	·	·	·
Lin and Chen^66^	·	·	·	￮	·	·	·	·	·	￮
Manganyi *et al*^28^^]^	·	·	￮	·	·	◐	·	·	·	·
Honarbakhsh *et al*^67^	·	·	￮	￮	·	·	·	·	·	·
Huh *et al*^68^	·	·	·	·	·	·	·	·	·	·
Foereland *et al*^69^	·	·	·	￮	N/A	·	·	·	·	·
Winski *et al*^70^	·	￮	￮	·	·	·	·	·	·	￮
Fakherpour *et al*^71^	·	·	·	·	·	·	·	·	·	·
Zhang *et al*^72^	·	·	·	·	·	·	·	·	·	￮
De‐Yñigo‐Mojado *et al*^73^	·	·	·	·	·	◐	·	·	·	·
Fakherpour*et al*^74^	·	·	·	·	N/A	·	·	·	·	￮
Williams *et al*^75^	·	·	·	·	N/A	◐	·	·	·	·

●Criteria met; ◐ criteria partially met; ◯ criteria not met.

Criteria: (1) were aims and objectives clearly stated? (2) was the study population clearly specified and defined? (3) were inclusion and exclusion criteria for being in the study prespecified and applied uniformly to all participants? (4) was a sample size justification, power description, variance or effect estimates provided? (5) were methods of anthropometric measurement clearly described, valid, reliable and implemented consistently across all study participants? (6) were other independent variables clearly defined, valid, reliable and implemented consistently across all study participants? (7) were the dependent variables clearly defined, valid, reliable and implemented consistently across all study participants? (8) is it clear what was used for analysis or to determine statistical significance estimates? (9) results—were basic data adequately described? (10) were limitations of study discussed?

Where indicated as ‘criteria not met’ for criteria, (3) inclusion and/or exclusion criteria have not been specified. Where indicated as ‘criteria not met’ for criteria, (4) no sample size justification or power calculation has been reported, nor assessment of variance or effect size. Most studies did not report sample size justification or power calculation, but criteria were deemed to be satisfied if variance or effect estimate provided.

Anthropometric measurements made from photographs of participants using established landmarks for five of seven facial dimensions. Protection factor scores required to pass not reported. Correlation analysis performed for only a white male subset of the study population.^48^

Correlation analysis for facial dimensions and respiratory protective equipment (RPE) fit reported as having been performed but results were not provided as no significant correlations made.^49^

The study was underpowered to assess for race.^50^

Facial measurements not entirely in keeping with standard anthropometric landmarks and measurements, as judged by included figure.^51^

Physical examination and pulmonary function performed but inclusion/exclusion criteria not stated.^29 52^

Study population not specified. Some participants did not test all respirator models and were substituted by others with similar face size categories.^54^

Once a successful fit test was obtained other models were not tested. The order of masks tested was applied consistently.^56^

Results of effect of gender, age and occupation reported only briefly.^57^

Data on facial categories collected rather than anthropometric measurements. Respirator for testing was selected by the tester based on observed facial characteristics rather than measured facial dimensions and Los Alamos National Laboratory categories. Once a successful fit test was obtained other models were not tested. Healthcare workers who failed fit testing were not tracked and if returned for second fit-testing sessions were treated as independent events.^59^

Two facial measurements were collected only on a small proportion of participants. SD provided but no between group comparisons available. Correlation analysis was not performed between the facial dimensions and fit factor.^61^

Once a successful fit test was obtained other models were not tested.^62^

Estimate of variance and/or effect size were irrelevant for aims of study to determine if RPE fit of respirator size relates to respirator fit test panel facial size categories.^64^

Participants that were not clean shaven were initially included in the analysis which likely skews results given known effect of facial hair on RPE performance.^28^

Factors such as facial hair presence was not records, and could influence the difference in fit factors between genders.^73^

Anthropometric data not collected. Ambiguous categorisation on ethnicity of participants as South East Asian and non-Asian.^75^

## Discussion

Our review demonstrates significant gender-based variance in standardised anthropometric measurements, with significantly smaller female FD for all measurements. Comparing Asian and black/African groups to Caucasians shows differences in facial geometry such as overall face size and nose measurements. With regard to RPE performance, female and BAME participants have generally low FF scores and/or fit-test PR. However, only a limited number of studies included BAME people in RPE fit-testing. Given the limited number of comparative studies available and heterogeneity in study design, we cannot be conclusive in our evaluation of RPE performance in gender or ethnic groups and their associations with specific anthropometric parameters.

BSI recognises anatomical and structural differences between genders.^77^ Our review shows that facial measurements included in RFTPs, namely face length, face width and lip width, are smaller for females. This is consistent with a large gender-based anthropometric study.^78^ In the context of fit-testing; most studies collected data limited to FD included in the LANL and NIOSH bivariate RFTPs. A limited number of studies collected additional facial measurements, such as nose dimensions, and showed that these features are relevant to RPE fit. Hence, the inclusion of these additional dimensions and their correlation to RPE performance would be valuable in future studies.

ISO has reported differences in facial characteristics between Caucasian, Sub-Saharan and European facial types.^77^ Comparisons between Caucasian and black participants demonstrate that the latter have greater protrusion of lips, greater head depth, and shorter, wider, shallower noses.^26 78^ Hispanic workers have significantly larger facial features for 14 measurements than Caucasians, with shorter nose protrusion and head length.^26^ Asian participants have statistically different dimensions as compared with Caucasians for 16 anthropometric values.^26^ However, only a limited number of studies comparatively evaluate the impact of ethnicity on RPE performance.

Furthermore, disaggregated comparisons are lacking for ethnicities outside predominant American groups (Caucasian, black, Hispanic). Often studies categorise participants as ‘Other’ which includes a diverse group of Central, South and East Asians, even though there are significant anthropometric differences between these groups based on ancestry.^79 80^ Our review also includes studies using American RFTPs as benchmarks, which show significant proportions of Chinese, Korean and Iranian participants’ facial measurements lie outside the distribution of American RFTPs.^66 71 81 82^ Additionally, individuals from Asian and black ethnic groups continue to be under-represented in RFTPs. There appears to be an urgent need to use fit-test panels that account for ethnicity-specific differences.

Gender-based anthropometric differences are associated with RPE performance in about half of our studies, the majority of which demonstrate that female participants have significantly lower RPE performance, need a variety of mask models for successful fit and are more likely to fail fit-testing altogether.^27–29 50 52 53 56 63 67 68^ The heterogeneity in results is likely related to study design, of which RPE availability and the assortment of models on offer are particularly relevant. First, many studies do not make gender-based comparisons of RPE performance for individual mask models, comparing overall fit-testing success between genders instead. This is based on successful fit-testing with at least one respirator, which fails to account for the higher fit-testing failure rates for individual RPE models among females, therefore reducing gender-based differences in RPE performance. Second, provision of one model in limited sizes or RPE designed as ‘one-size-fits-all’ fails to cater to smaller FD. Increasing RPE choice improves user success rates and reduces gender-based fit-testing differences. For example, a study demonstrated that inclusion of two additional models accounts for a 20% improvement in female PR.^54^ Certainly, several studies included here recommend a variety of RPE should be made available to ensure successful fit-testing.^30 56 58 61 62 65 69 71 74^ In practice, implementing a comprehensive fit-testing programme is a financial and logistical challenge.^59^ The variety of RPE in different healthcare environments is variable and procurement dependent. It may not be feasible to test HCWs on all available RPE given the time-consuming nature of fit-testing.

Studies report mixed results for ethnicity-based differences in RPE performance. Small comparative studies have demonstrated lower PR for black and Asian females, but with no effect of ethnicity on FF scores.^28 51 61^ These studies were likely underpowered to recognise subgroup differences. Studies of Asian populations have consistently yielded higher rates of fit-test failure among Chinese, Koreans, Taiwanese and Iranians, further emphasising the need to consider FD of their population in RPE design.^29 52 53 63 66–68 71 72 74^ Therefore, RPE currently available does not provide comparable protection across ethnicities, likely disadvantaging those from minority groups. This implies, RPE design may be failing to accommodate for heterogeneity in facial features across diverse user populations due to the limited panels used for international standards in their manufacture.

The 2007 NIOSH updated panel and 2014 ISO standards (ISO 16900-1:2014) aim to reflect greater end user diversity. While efforts to diversify panels have been promulgated, many respirators in current use meet outdated standards from early 2000s (EN 149:2001+A1:2009) which comprise a very limited panel. This is supported by a survey of FFP3 respirators used across acute NHS centres during the COVID-19 pandemic.^83^ Therefore, designing RPE that fit a wide range of demographics is difficult if RPE is permitted to satisfy standards with limited representation.

In practice, poorly fitted RPE hamper work and user safety.^84 85^ Widespread concerns around inadequacies in areas of RPE fit-test access, availability and training have been raised.^86 87^ Unfortunately, the proportion of female and BAME HCWs affected and the need for personalised RPE has not been quantified.^85^ Studies included in this review were not designed to identify modifications during RPE donning, such as excessive tightening of straps or use of adhesive tape which may allow for successful fit-testing but indicate poor RPE fit. Notably, skin damaged is reported to affect 42%–97% of HCWs and ill-fitting RPE may account for higher rates of adverse reactions among BAME HCWs.^83 88–90^ Given the lack of data, specific guidance on modification measures are limited from NHS England and NHS Improvement.^91^ Modifications during RPE donning many affect RPE efficacy and the presence of facial lesions encourage face touching and mask handling, resulting in inadvertent PPE contamination.^92–97^

### Strengths and limitations

This is the first systematic review and meta-analysis of the influence of gender and ethnicity on RPE, to the best of our knowledge. Our search strategy and eligibility criteria were broad and have captured a large number of relevant studies. However, we were limited to English-based databases. We excluded studies in Chinese as we were unable to gain access to the data. This is a significant limitation considering the focus of our review and inclusion of non-English studies may affect results significantly.

Inherent associations exist between gender and FD as well as multicollinearity between FD, although these associations were not always clearly accounted for or reported by studies. Meta-analysis showed significant heterogeneity existed for nine FD. Of these measurements, those with small magnitude of effect (ie, smaller differences in measurements) such as nasal root breadth (MD 0.37 mm), nose length (MD 3.64 mm), nose protrusion (MD 2.03 mm) and lip width (MD 2.82 mm) may be less relevant or irrelevant to gender-based differences in anthropometrics. By extension, they may be less relevant to RPE fit.

There was significant disparity in study design and methodology in gender-based studies. Assessment of study design confirmed anthropometrics were collected by standardised methods. Only one study reported conflicting results, with FD greater for females. Exclusion of this study did not sufficiently improved heterogeneity. BAME people have different FD to Caucasians, and it was suspected that heterogeneity may be result of participant diversity. However, subgroup analysis based on ethnicity was not possible as studies measured varying combinations of FD and ethnicity-based grouping reduced sample size such that meta-analysis would not provide meaningful conclusions. Risk of bias assessment demonstrated most studies failed to meet criteria three, relating to use of prespecified inclusion and exclusion criteria. This may contribute to heterogeneity observed in meta-analysis of anthropometrics and differences in conclusions regarding gender-based differences in RPE performance. Several studies failed to account for their sample size through justification, power calculation or estimate of variance/effect. These risks studies being underpowered to detect differences in RPE performance between gender and/or ethnic groups, and may account for the conflicting results. Limited number of studies included ethnically diverse participants with all relevant anthropometrics. Hence, we cannot be conclusive in our evaluation of RPE performance on gender or ethnic groups and their associations with specific anthropometric parameters.

### Future research

Successfully fit-testing HCWs is particularly important in the current climate. Future studies addressing the disparity in RPE fit will require a review of how respirators are designed and tested, including use of a relevant fit-test panel. Studies should aim to include a diverse group of participants inclusive of BAME people to better inform future mask design and fit testing performance. Studies should include the provision of a variety of mask models, brands and sizes, denoting modifications made during the donning process, and the fit-test PR for all mask models tested rather than using an overall success rate. Longitudinal studies on how facial anthropometrics influence fit, but also user comfort and adverse outcomes thereafter would be useful to inform mask designs. The future clearly lies in personalising fit-testing with modern technology. For example, three-dimensional facial model-capture may be used to assess fit in order to reduce time and costs of fit-testing as well as expedite identification of HCWs who need alternative RPE.

## Conclusion

Anthropometric data is key in the design and testing of respirators, and user demographics reflected in respiratory fit test panels may influence the level of protection respirators provide. Facial measurements vary significantly between gender and ethnicity. Our meta-analysis demonstrates women have significantly smaller facial measurements for 14 standardised measurements compared with men. The literature suggests significant differences in anthropometrics between ethnicities, however, minority groups continue to be under-represented in comparative studies and race-based differences could not be established in our study. The effect of differences in facial anthropometrics on respirator fit and effectiveness is less clear. Over half of studies reporting gender-based comparisons in RPE performance report significantly lower PR among females. Three studies report lower PR among Asian or black participants. However, these PR differences are inconsistently associated with absolute FF scores. FD across ethnic minorities may fall outside the parameters of current RFTPs and impact RPE performance. Therefore, RFTPs need to be expanded to capture the distribution of anthropometric data from all ethnicities and RPE development needs to reflect a more diverse group of users.

## Data Availability

All data relevant to the study are included in the article or uploaded as supplementary information.
