# Supplementary material for: The influence of gender and ethnicity on facemasks and respiratory protective equipment fit: a systematic review and meta-analysis
Source: BMJ Glob Health. 2021 Nov 11;6(11):e005537. doi: 10.1136/bmjgh-2021-005537 (PMC8587533; doi:10.1136/bmjgh-2021-005537)
Supplement: Supplementary data [file bmjgh-2021-005537supp002.pdf]

## Appendix 2. Search Strategy

Ovid – Embase search

1. exp minority group/
2. ethnicity/ or exp ethnic group/
3. race difference/ or race/
4. (BME or BAME).mp.
5. South Asian/ or Southeast Asian/ or Central Asian/ or Asian continental ancestry group/ or West Asian/ or Asian American/ or British Asian/ or Asian/ or East Asian/
6. Black person/
7. African American/ or African Caribbean/ or African/ or East African/ or West African/ or North African/ or South African/
8. 1 or 2 or 3 or 4 or 5 or 6 or 7
9. anthropometry/ or face/ or facial measurements.mp.
10. facial dimensions.mp.
11. 9 or 10
12. 8 or 11
13. mask.mp. or face mask/ or mask/
14. (face mask$ or facemask$ or respirator$).mp.
15. Personal Protective Equipment.mp. or protective equipment/
16. Respiratory Protective Devices.mp.
17. Respiratory Protective Equipment.mp.
18. (RPE or PPE).mp.
19. Face protect$.mp.
20. 13 or 14 or 15 or 16 or 17 or 18 or 19
21. fit test$.mp.
22. mask fit$.mp.
23. respirator fit$.mp.
24. 21 or 22 or 23
25. 12 and 20 and 24
