# Supplementary material for: The influence of gender and ethnicity on facemasks and respiratory protective equipment fit: a systematic review and meta-analysis
Source: BMJ Glob Health. 2021 Nov 11;6(11):e005537. doi: 10.1136/bmjgh-2021-005537 (PMC8587533; doi:10.1136/bmjgh-2021-005537)
Supplement: Supplementary data [file bmjgh-2021-005537supp003.pdf]

Appendix 3. Reason for exclusion of records at full-text review

Studies	*Reason for exclusion*
Cherrie et al. (2018) [36]	• Masks assessed also included cloth masks<br>• Primary effectiveness testing did not involve fit-testing using human participants.<br>• Only 2 of 5 filtering face-piece half masks were tested on participants
Coffey et al. (2010) [37]	• Comparison of fit-testing methods to assess which test methods best identify poorly fitting respirators
Coffey et. (2004) [38]	• Assessment of four performance measures were used to evaluate the fitting characteristics of 18 models rather than effect of anthropometrics/gender/race on fit-testing outcomes
Fakherpour et al. (2021) [39]	• Advanced publishing full text without figures for review of results
Han and Lee (2005) [40]	• Comparison of Inward Leakage and Total Inward Leakage testing<br>• Some respirators used which do not meet the FFp3/N95 level of protection
Han et al. (2018) [41]	• Assessment of a respirator modification, a tight-fitting net, to improve facial fit and respirator performance<br>• Details of the filtering facepiece masks used not reported<br>• No assessment of N95/FFP3 masks in the context of anthropometrics/gender/ethnicity
Hui et al. (2017) [42]	• Assessment of a novel mouldable filtering facemask performance as compared to standard respirators<br>• No assessment of N95/FFP3 masks in the context of anthropometrics/gender/ethnicity
Lawrence et al. (2006) [43]	• Comparison of elastomeric facepiece masks, filtering facepiece masks and surgical masks performance<br>• No assessment of N95/FFP3 masks in the context of anthropometrics/gender/ethnicity
Regli and Ungern-Sternberg (2020) [44]	• Perspectives piece<br>• No original research data
Reponen et al. (2011) [45]	• Assessment of value of fit-testing and its effect on respirator protection<br>• No assessment of N95/FFP3 mask performance in the context of anthropometrics/gender/ethnicity
Sietsema and Brosseau (2018) [46]	• Studied whether quantitative fit factors are predictive of respirator fit during simulated activities<br>• No assessment of N95/FFP3 mask performance in the context of anthropometrics/gender/ethnicity
Suen et al. (2020) [47]	• Comparison of mask fit and usability of two types of respirators in the context of nursing procedures<br>• No assessment of N95/FFP3 mask performance in the context of anthropometrics/gender/ethnicity
