# Supplementary material for: The influence of gender and ethnicity on facemasks and respiratory protective equipment fit: a systematic review and meta-analysis
Source: BMJ Glob Health. 2021 Nov 11;6(11):e005537. doi: 10.1136/bmjgh-2021-005537 (PMC8587533; doi:10.1136/bmjgh-2021-005537)
Supplement: Supplementary data [file bmjgh-2021-005537supp004.pdf]

Appendix 4. Distribution of participants across gender and ethnic groups

Population characteristic	*Participants*					
	Mixed population studies (n=8)		*Single population studies (n=16)*		*All studies (n=32)*	
**Total participants**	6,068		2,477		10,658	
**Male**	1404	(23%)	1,414	(57%)	3,477	(33%)
**Female**	4,163	(69%)	1063	(43%)	6,351	(60%)
**Not reported**	501	(8%)	-	-	830	(8%)
**White**	4,710	(78%)	399	(16%)	5,109	(48%)
**African/Black**	456	(8%)	-	-	456	(4%)
**Hispanic/Latino**	65	(1%)	56	(2%)	121	(1%)
**Asian**	390	(6%)	1,639	(66%)	2,029	(19%)
**South/Central Asian**	177	(3%)	383	(15%)	560	(5%)
**Aboriginal**	43	(1%)	-	-	43	(<1%)
**Mixed race/coloured**	55	(1%)	-	-	55	(1%)
**Other**	55	(1%)	-	-	55	(1%)
**not reported**	71	(2%)	-	-	2,230	(21%)
