# Supplementary figures and images for: The influence of gender and ethnicity on facemasks and respiratory protective equipment fit: a systematic review and meta-analysis
Source: BMJ Glob Health. 2021 Nov 11;6(11):e005537. doi: 10.1136/bmjgh-2021-005537 (PMC8587533; doi:10.1136/bmjgh-2021-005537)

## Appendix 5.

## Bicteoorbitale breadth

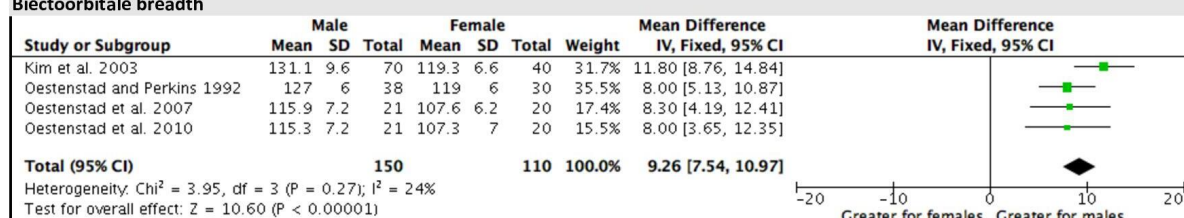

## Bizygomatic breadth [Face width]

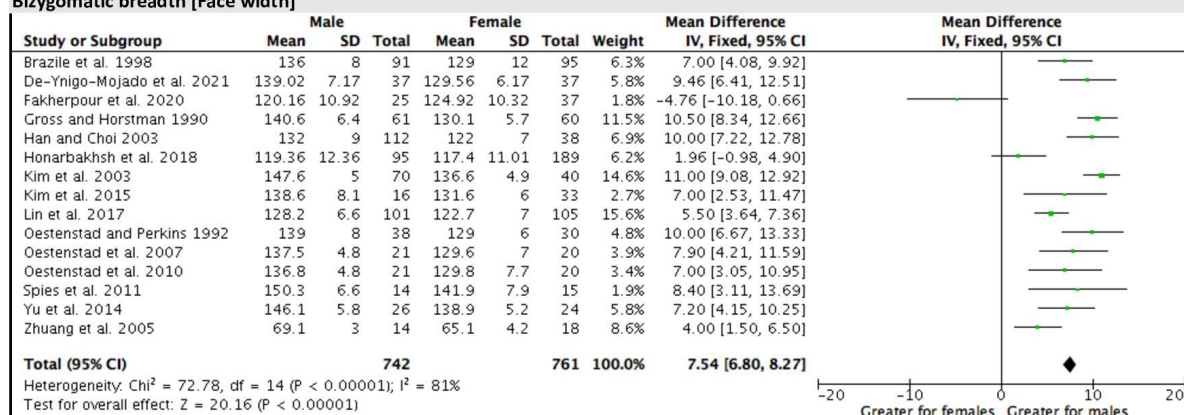

Supplement: Supplementary data [file bmjgh-2021-005537supp005.pdf]
