# Supplementary material for: The influence of gender and ethnicity on facemasks and respiratory protective equipment fit: a systematic review and meta-analysis
Source: BMJ Glob Health. 2021 Nov 11;6(11):e005537. doi: 10.1136/bmjgh-2021-005537 (PMC8587533; doi:10.1136/bmjgh-2021-005537)
Supplement: Supplementary data [file bmjgh-2021-005537supp006.pdf]

Appendix 6. Association of variables facial dimensions, gender and ethnicity with RPE fit

Studies	*Pass rates (PR) Users/RPE groups**	*Association between facial dimensions (FD) and fit***	*Association between gender and fit***	*Association between ethnicity and fit***	*Association of mask factors****	*Recommendations or conclusions made by authors*
Liau et al. (1982) [48] US	N/A	low correlation coefficients but greatest for FW and LW	N/A	N/A	N/A	No FD recommendation
Gross and Horstman (1990) [49] US	Users: 95%<br>♂: 83-85%<br>♀: 71-89%<br>(RPE dependent)	Nose and lip measurements were relevant but no significant correlation	PR for 2/3 RPE models: ♂ > ♀<br>Comparable PR between genders with >1 RPE models	N/A	variability of FF based on brand for ♀	No FD recommendation. Need to offer variety in RPE model and size.
Oestenstad et al. (1990) [50] US	N/A	High prevalence of nose leaks. Significant correlation for NRB, BIOC, LFL to FF.	Association of facial dimension and leak sites attributed to gender. Greater predictability with gender specific models.	N/A	N/A	Consideration for inclusion of nasal dimensions
Oestenstad and Perkins (1992) [27] US	Users: 80.9%	FDs predict fit.<br>LFL alone is a good predictor. FL and NRB are also relevant	Gender based differences in fit predicted by differences in FD. Greater predictability with race/gender specific models	Race specific models improve predictive ability	N/A	Other FDs (i.e. LFL) may be more appropriate
Brazile et al. (1998) [51] US	Users: 91.9%<br>♂: 91.2%<br>♀: 92.6%	NW and NP were associated with FF but account for small percentage of FF variation and were not good predictors of fit	No significant association<br>Comparable FF and PR between genders	Black♀:<br>lowest PR<br>No effect on FF	N/A	Fit is associated with individual FD rather than FD associated with gender or ethnicity
Han (2000) [52] South Korea	RPE groups:<br>30.9 - 77.2%	Significant FF variation between facial size categories	Difference in FF scores: ♂ > ♀	Low/moderate PR amongst Korean users ‡	Significant difference in FF and PR between brands	Respirators designed for males may not fit females. FL and LW alone are not good criteria
Han and Choi (2003) [29] South Korea	♂: 58 - 78.6%<br>♀: 34.2 - 50%<br>(RPE dependent)	FW, NP and BTMA arc are good predictors of fit. No common FD variable across all RPE/gender subgroups	Difference in PR for 2/3 RPE models: ♂ > ♀<br>Gender-specific models account for most variation in fit factor	Low/moderate PR amongst Korean users ‡	Significance difference in FF and PR between brands	The 3 FD predictive of fit should be considered in designing RPE for Koreans
Kim et al. (2003) [53] South Korea	♂: 41.4 - 87%<br>♀: 10.3 - 60%<br>(RPE dependent)	♂: No correlation for FD and FF<br>♀:5 FD correlate with FF (BTSA, BECTO, BTMA, JW, LFL)<br>FW, LFL, BIOC, BECTO, NL, LW, BTSA predict PR/fit (gender/RPE dependent)	Difference in FF and PR: ♂ > ♀<br>Greater predictability with gender specific models	Low/moderate PR amongst Korean users ‡	Significance difference in FF and PR between brands	Consideration of alternative FD in design of quarter mask respirators for Koreans
Zhuang et al. (2005) [54]	N/A	16/33 models of RPE model/size combinations show association of	Comparable FF between genders for 16/18 RPE models	N/A	FF associated with number of sizes per	FL and FW are recommended for defining the panel
